# Comparative Accuracy of Stationary and Smartphone-Based Photogrammetry in Oral and Maxillofacial Surgery: A Clinical Study

**DOI:** 10.3390/jcm13226678

**Published:** 2024-11-07

**Authors:** Lukas B. Seifert, Michel Beyer, Vanessa Czok, Alexander Aigner, Sead Abazi, Florian M. Thieringer, Robert Sader

**Affiliations:** 1 Department of Oral, Cranio-Maxillofacial and Facial Plastic Surgery, University Hospital Frankfurt, Theodor-Stern-Kai 7, 60528 Frankfurt am Main, Germanyrobert.sader@kgu.de (R.S.); 2 Clinic of Oral and Cranio-Maxillofacial Surgery, University Hospital Basel, CH-4031 Basel, Switzerland; michel.beyer@usb.ch (M.B.); alexander.aigner@usb.ch (A.A.); sead.abazi@usb.ch (S.A.); florian.thieringer@usb.ch (F.M.T.); 3 Medical Additive Manufacturing Research Group (Swiss MAM), Department of Biomedical Engineering, University of Basel, CH-4123 Allschwil, Switzerland

**Keywords:** 3D scanning, facial scanning, smartphone

## Abstract

**Background:** Three-dimensional facial scan technologies, such as stereophotogrammetry, are frequently used in oral and maxillofacial surgery, dentistry, and plastic surgery to assess patient outcomes and plan surgical procedures. Most facial scanners rely on stationary clinical systems, which provide high accuracy and reliability in generating 3D representations of the human face; however, they are cost-intensive and immobile. Recently, smartphone-based facial scan applications that use stereophotogrammetry have been developed. These applications are cost-effective and more accessible than existing stationary systems. **Methods:** In this study, we compared the accuracy of three different smartphone applications—EM3D, Polycam, and ScandyPro—on an iPhone 14 Pro, which utilizes Apple’s LiDAR (Light Detection and Ranging) technology, to a stationary system by 3DMD, which is considered a gold standard in many studies. **Results:** The applications included in the study have demonstrated the capability to perform accurately in clinical settings, with mean surface distances of 1.46 mm for EM3D, 1.66 mm for Polycam, and 1.61 mm for ScandyPro. **Conclusions:** These results suggest that smartphone-based facial scan systems could be a viable alternative, particularly in resource-limited settings. However, further research and collaboration between academia and industry are necessary to fully integrate these technologies into clinical practice.

## 1. Introduction

The application of facial scan technologies has significantly advanced in recent years and is increasingly being utilized in various medical fields, such as oral and maxillofacial surgery, dentistry, and plastic surgery. Traditionally, stationary clinical systems have been regarded as the gold standard for creating precise and detailed three-dimensional representations of the human face. These systems are characterized by their high accuracy and reliability, making them indispensable for surgical planning, diagnostic purposes, and postoperative evaluations [1]. Among others, computed tomography (CT) and cone beam computed tomography (CBCT) are prominent techniques known for their high-resolution images and ability to capture both hard and soft tissues. CT and CBCT are widely used in maxillofacial and dental imaging due to their precision, but they expose patients to ionizing radiation, which is a significant drawback [2,3]. Additionally, the high cost and limited accessibility of these systems can be a barrier in some clinical settings. Laser-based scanning systems, such as laser triangulation and structured light systems, offer high accuracy and resolution. These systems work by projecting a laser beam onto the face and capturing the reflected light to create a detailed 3D model. The main advantages of laser scanning are its precision and the ability to capture fine details of the facial surface. However, these systems can be expensive and require specialized equipment and training to operate effectively [4,5]. Another technique, photogrammetry, involves capturing multiple two-dimensional images from different angles and using specialized software to reconstruct a three-dimensional model. This method is non-invasive and relatively cost-effective and hence can be found in most modern face scan systems today. However, its accuracy can be influenced by lighting conditions and the quality of the camera used [6].

Recently, smartphone-based facial scan applications have made significant strides, offering a more cost-effective and accessible alternative to stationary clinical systems. These mobile technologies leverage the advanced hardware features of modern smartphones, such as Apple’s LiDAR (Light Detection and Ranging) technology, which projects and analyzes thousands of invisible dots to create a precise depth map of the face. This technology allows for highly accurate 3D facial recognition and scanning, enhancing the precision of photogrammetry-based scans [7]. While stationary systems are considered the gold standard due to their established use and validation, smartphone-based systems offer the advantages of mobility and lower costs, making them particularly attractive for smaller practices or use in developing countries [8].

The aim of this study, therefore, is to compare and evaluate the accuracy of stationary clinical photogrammetry systems and smartphone-based facial scan systems, and to determine the extent to which smartphone-based systems can represent a clinically reliable alternative.

## 2. Materials and Methods

### 2.1. Study Participants and Study Conduction

This study was conducted at the University Hospital Frankfurt in the Department of Oral, Cranio-Maxillofacial, and Facial Plastic Surgery and at the University Hospital Basel in the Department of Oral and Cranio-Maxillofacial Surgery. The study population consisted of 15 healthy volunteer participants (6 males, 9 females, mean age = 25 years). Participants with excessive facial hair were excluded from the study, and female participants were requested not to wear makeup. All participants provided written informed consent to participate in the study, which could be withdrawn at any time. The study adhered to the principles of the Declaration of Helsinki, and no further review by the local ethics committee was required.

The facial scans were performed in a single day for each participant. The stationary photogrammetry system from the company 3dMD (3dMD Research Limited, Wexford, Ireland) was used as the gold standard since previous studies have found high accuracy regarding the generated face scans [7]. This system was calibrated according to the manufacturer’s specifications prior to image capturing. Participants were seated approximately 1.5 m from the front of the facial scanner. Six cameras, positioned at different angles, captured an image sequence, which was then rendered into a 3D mesh using the scanner’s software. This 3D mesh was subsequently exported as an .obj file.

Following this, three additional face scans were conducted for each study participant using an iPhone 14 Pro (Apple, Cupertino, CA, USA), priced at USD 1000 at launch, with the 3D scan apps EM3D (Version 1.0, Brawny Lads Software, LLC, Cincinnati, OH, USA), Polycam (Version 3.0, Polycam Inc., San Francisco, CA, USA), and ScandyPro (Version 2.1.4, Scandy LLC., New Orleans, LA, USA), each utilizing the iPhone’s LiDAR (Light Detection and Ranging) sensor. These apps were selected primarily based on their cost-effectiveness: EM3D and Polycam were available free of charge, while ScandyPro offered an affordable option at EUR 1.99 per week. LiDAR technology emits pulses of infrared light and measures the time it takes for the light to return after reflecting off objects. This allows the sensor to generate a precise 3D map of the environment [9].

To create the 3D face scans, the smartphone was mounted on a tripod. Participants were seated on a rotating stool approximately 1 m away from the tripod and rotated 360°, generating a 3D scan that could be exported as an .obj file. The scanning process took approximately 40 s per scan after familiarization. All scans were consistently performed under controlled lighting conditions, specifically excluding daylight. The face of each scanned individual was uniformly illuminated from all directions using overhead lighting, ensuring consistent and reproducible image capture conditions across all scans. 

**Figure 1:**
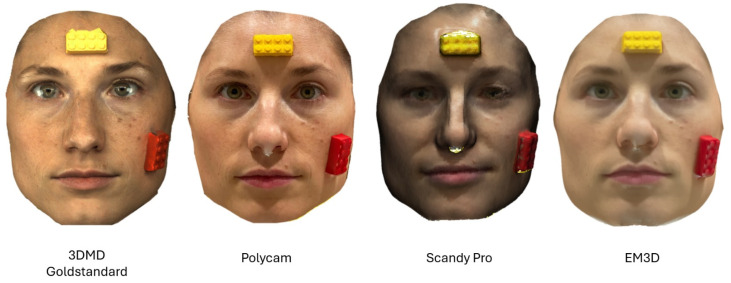
Texture comparison for each facial scanner of participant Nr. 1.

To enable post-scan scaling of the dimensionless scans, a Lego brick was attached to the forehead and the left cheek of each participant using a glue stick (Figure 1). The Lego bricks were chosen due to their precise, reproducible geometry and dimensions, making them ideal for standardized testing. Additionally, their use in previous studies on this topic [10] further supports their reliability as reference objects in accuracy assessments of low-cost facial scanners. The exact dimensions of the Lego bricks were measured beforehand using calipers. After exporting the 3D scans as .obj files, they were imported into the open-source software Meshlab (Version 2022.02, Visual Computing Lab, Pisa, Italy) [11] and scaled to the original size of the Lego bricks. Additionally, the scans were digitally cropped to the facial region of the participants, which was used for the subsequent accuracy analysis.

**Figure 2:**
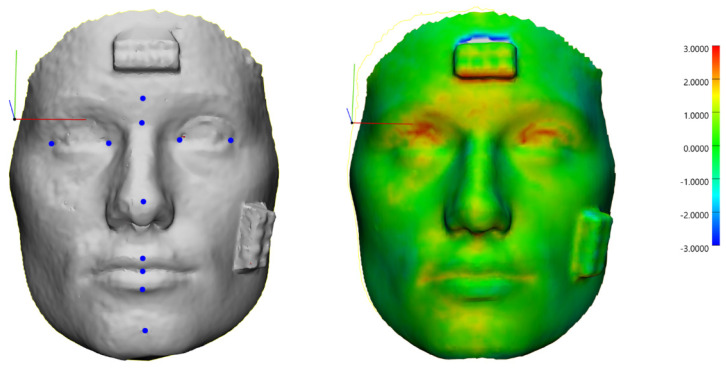
Three-dimensional facial scans registered onto the 3dMD system using the Interactive Closest Point (ICP) algorithm, with landmarks marked and color-coded surface deviations visualized.

The scaled scans were imported into Materialise 3-Matic (Version 17.0, Materialise NV, Leuven, Belgium), where they were registered onto the 3dMD scan using an Interactive Closest Point (ICP) algorithm. Subsequently, the landmarks listed in Table 1 were marked onto each scan by two independent raters (Figure 2).

### 2.2. Accuracy Analysis

To assess the accuracy, the surfaces of the scans were compared to the surface of the ground truth, and the mean surface distance (MSD) and maximal distance (MaxD) were calculated. The formulas for these metrics are displayed in Table 2. Additionally, for each landmark, the average distance to its corresponding landmark on the ground truth scan was calculated.

### 2.3. Statistical Analysis

To assess the consistency of measurements between the two raters, the intraclass correlation coefficient (ICC) was calculated for each landmark.

To account for the repeated measurements of 9 facial landmarks on each participant, we adjusted the sample size calculation using the software G*Power (University of Düsseldorf, Düsseldorf, Germany). An intraclass correlation coefficient (ICC) of 0.5 was assumed between the scanners, and a Bonferroni correction was applied to account for multiple comparisons across the 9 landmarks, adjusting the alpha level to 0.0056. Using the G*Power software (version 3.1.9.7, Heinrich Heine University Düsseldorf, Düsseldorf, Germany), we performed the sample size calculation with the following parameters: a medium effect size (Cohen’s d = 0.5), an alpha level of 0.0056, a power of 0.8, and two-sided testing. The adjusted calculation showed that 106 data points were needed, and since 405 points were collected across all the tested scanners, the sample size was considered to be sufficient to detect significant differences between the scanners.

Statistical analyses were conducted to assess the distribution and differences among measurements from three face scanners. The Shapiro–Wilk test was employed to evaluate the normality of the data, revealing that most measurements were not normally distributed. Consequently, the Kruskal–Wallis test was utilized to compare the median deviations of landmark measurements across the scanners. A *p*-value of less than 0.05 was considered indicative of significant differences between the groups.

### 2.4. AI Usage

ChatGPT (version 4.0) was utilized exclusively for the improvement of sentence structure and language flow. No content generation, substantive analysis, or intellectual contributions were made by the AI system beyond linguistic enhancement.

## 3. Results

### 3.1. Surface Comparison

The results of the surface comparison between the scanner apps and the ground truth are displayed in Table 3. The mean surface distance amounts to 1.46 mm for the EM3D, 1.66 mm for the Polycam, and 1.61 mm for the SP scanner, whereas the maximal distance amounts to 16.61 mm for the EM3D, 18.81 mm for the Polycam, and 21.82 for the SP scanner.

### 3.2. Landmark Comparison

**Figure 3:**
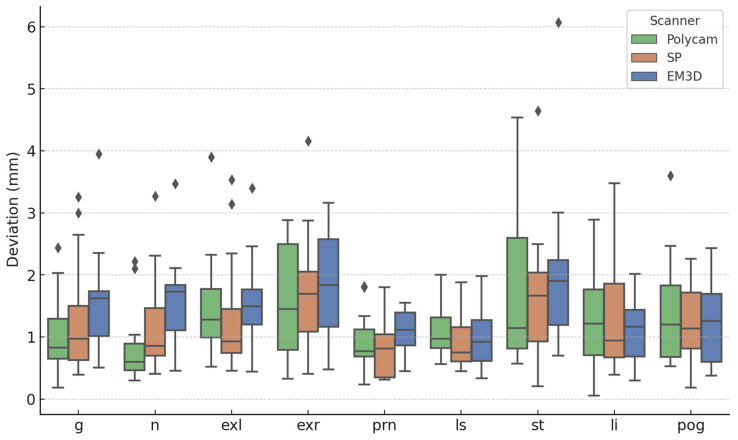
Distances between the landmarks (averaged between rater 1 and rater 2) of the scanners and the ground truth (glabella—g; nasion—n; exocanthion left—exl; exocanthion right—exr; pronasale—prn; labius superius—ls; stomion—st; labius inferius—li; pogonion—pog). The diamonds represent outliers in the data (values more than 1.5 times above or below the interquartile range).

The results of the landmark comparisons are displayed in Table 4 and represented graphically in Figure 3. The mean distance of all the landmarks amounts to 1.27 mm for the Polycam, 1.26 mm for the SP, and 1.45 mm for the EM3D scanner. The intraclass correlation coefficient (ICC) was calculated for each anatomical landmark, yielding a mean ICC of 0.807, indicating a high level of measurement consistency across the raters. Finally, the ANOVA analysis between each scanner for every landmark showed no significant differences between the tested scanners (Table 5).

## 4. Discussion

The aim of this study was to compare the accuracy of a stationary clinical photogrammetry system with smartphone-based scan applications. The results of the assessments showed no statistically significant differences between the scanners. The smartphone applications tested in this study (EM3D, Polycam, and ScandyPro) demonstrated clinically acceptable performance, with MSD values of 1.46 mm, 1.66 mm, and 1.61 mm, respectively. These findings indicate that smartphone-based systems, leveraging technologies such as Apple’s LiDAR, can produce clinically relevant 3D facial scans.

In addition to the surface comparisons, our study also performed a detailed analysis of specific anatomical landmarks. The results of the landmark comparison revealed that the accuracy of each smartphone-based application varied slightly across different facial regions, but overall, the performance remained within clinically acceptable ranges. Notably, the ScandyPro application demonstrated the smallest overall mean distance deviation of 1.26 mm across all landmarks, followed closely by Polycam with 1.27 mm, and EM3D with 1.45 mm.

When examining individual landmarks, we observed that the nasion (n) consistently showed the lowest deviations across all applications, with Polycam achieving the smallest deviation of 0.82 mm, while EM3D showed a slightly higher deviation of 1.58 mm. It was also observed in the Kruskal–Wallis test that the nasion was the only point where statistically significant results were found between the scanners (*p* = 0.0128). The nasion lies at the intersection of various facial planes, making it more challenging for smartphone-based algorithms to accurately capture its position compared to other landmarks. Additionally, variations in the algorithms used by different smartphone applications could also contribute to these discrepancies. Conversely, the largest deviation was observed at the stomion (st) for all applications, with deviations ranging from 1.65 mm for ScandyPro to 2.02 mm for EM3D. This finding suggests that landmarks located in more flexible or variable regions of the face, such as the mouth, may be more challenging to capture accurately with smartphone-based scanning applications compared to more rigid landmarks like the nasion or glabella (g).

The intraclass correlation coefficient (ICC) analysis demonstrated strong consistency between raters across all landmarks, with an overall ICC of 0.807. This suggests a high level of reproducibility in landmark identification, further supporting the reliability of smartphone-based systems.

These results highlight that, while some minor discrepancies exist between different applications, all three smartphone-based systems provide clinically acceptable results for landmark accuracy.

The observed differences between surface and landmark comparisons are likely due to the nature of the regions being measured. Surface analysis averages variations across the entire face, while landmarks focus on specific points, where movement or soft tissue flexibility—especially in areas like the mouth—can cause larger deviations. Additionally, smartphone-based scanners, despite using advanced LiDAR technology, may still struggle with capturing fine details in mobile or complex facial regions, leading to discrepancies between surface and landmark accuracy.

Previous studies have also investigated the accuracy of smartphone-based facial scanners [12,13,14,15]. Ettore et al. compared three-dimensional facial scans obtained by stereophotogrammetry with two smartphone applications that support the TrueDepth system and found that processing times with smartphone applications (Bellus3D and Capture) were considerably longer compared to the gold standard scanner (3dMD system). They reported an average mean surface distance of 1.0 mm and a standard deviation of 0.5 mm [13]. Another study by Van Lint et al. compared the accuracy of three-dimensional scans captured with a clinically approved portable stereophotogrammetry device (3D Vectra H1 Camera) to scans obtained on an iPad Pro using five smartphone applications (Hedges 3D Scanner, Bellus FaceMaker, ScandyPro, Scaniverse, and Trnio). They found the largest linear deviations with the Hedges 3D Scanner (3.4 ± 1.5 mm) and ScandyPro (4.4 ± 2.1 mm), while the smallest deviation was observed with the Bellus FaceMaker (2.2 ± 1.2 mm). Interestingly, our study found smaller deviations for the ScandyPro application (1.61 ± 0.42 mm), which might be due to the use of the 3DMD scan system as the gold standard in our study. A direct comparison between the 3DMD system and the 3D Vectra H1 using previously measured anthropometric landmarks might be the subject of future studies to determine the accuracy of both systems. Lastly, a study by Akan et al. [14] compared the 3DMD face scan system with a smartphone-based application (Bellus 3D) on an iPhone X and found statistically significant differences in the distances between the inner commissures of the right and left eye fissures and the nasolabial angle. The statistically significant differences in accuracy could be explained by the use of an older generation iPhone that did not utilize Apple’s LiDAR (Light Detection and Ranging) technology, resulting in less accurate face scans. The mean deviation of all the smartphone-based scan applications in this study was in the range of 1.0–2.0 mm for both surface and landmark comparisons. These results indicate that stationary systems, such as the 3DMD system, remain the gold standard, but smartphone-based scan applications can produce clinically relevant 3D face scans. The portability and cost-effectiveness of these systems present significant advantages over stationary systems, simplifying clinical work. These systems could be especially useful in resource-limited settings, such as smaller practices or in developing countries, where access to expensive systems may be restricted. Furthermore, the convenience of smartphone-based scanning allows for more frequent and accessible monitoring of patients’ conditions without the need for specialized equipment or extensive training. In the future, as smartphone technology continues to improve, there will be significant potential for integrating simple solutions like the ones presented in this study into routine clinical practice. This could lead to a wider adoption of modern technologies, reduced healthcare costs, and improved clinical outcomes for patients. Including patients with facial asymmetry or undergoing reconstructive surgery could also improve precision assessment and evaluate measurement accuracy in more complex clinical cases, further supporting the integration of these technologies in diverse clinical scenarios.

Lastly, while this study highlights the accuracy and cost-effectiveness of smartphone applications, further exploration is needed to fully assess their practical feasibility, particularly in resource-limited settings. The cost of devices like the iPhone 14 Pro, which is integral to the scanning process, could be a significant barrier to widespread clinical implementation in these regions. Additionally, integrating these technologies into routine clinical workflows may require additional training for clinicians. Addressing these challenges will be crucial for the successful adoption of these tools in diverse healthcare settings.

## 5. Limitations and Strengths

The number of participants (*n* = 15) may limit the statistical power of this study. However, this study is the first to compare smartphone-based 3D scan applications using Apple’s LiDAR (Light Detection and Ranging) technology with the 3DMD system, which is considered the gold standard in many studies for capturing three-dimensional scans of the human face.

## 6. Conclusions

In summary, our results suggest that 3D scan applications using the latest generation of smartphones offer a clinically viable, portable, and cost-effective alternative to stationary systems for acquiring three-dimensional images of the human face.

## Figures and Tables

**Table 1:** Landmark definitions.

Name	Abbreviation	Definition
Glabella	g	Most noticeable midpoint between the eyebrows
Nasion	n	Lowest point of the nasal bridge
Exocanthion Left	exl	Outer corner of the left eye fissure
Exocanthion Right	exr	Outer corner of the right eye fissure
Pronasale	prn	Most protruding point of the nose apex
Labiale Superius	ls	Midpoint of the upper lip line
Stomion	st	Point where upper and lower lips meet on the midline
Labiale Inferius	li	Midpoint of the lower lip line
Pogonion	pog	Most prominent point of the chin along the midline

**Table 2:** Metrics with their corresponding formula and legend.

Metric	Formula	Legend
Mean Surface Distance (MSD)	$MSD=\frac{1}{n_{A}}\sum_{i=1}^{n_{A}}\;\underset{p\in P}{min}\;∥a_{i}-p∥$	The average distance between points on the test surface and the closest points on the reference surface. Lower values indicate better overall alignment.
Maximal Distance (MaxD)	$MaxD=\underset{i=1}{\overset{n_{A}}{max}}\;\left(\underset{p\in P}{min}\;\left(∥a_{i}-p∥\right)\right)$	The largest single distance between any point on the test surface and its closest point on the reference surface. A smaller value means the worst deviation is minimal.

**Table 3:** The results of the surface comparison by describing the mean surface distance (MSD), the maximal distance (MaxD), and the standard deviation (SD).

	EM3D (mm)	Polycam (mm)	SP (mm)
MSD ± SD	1.46 ± 0.60	1.66 ± 0.97	1.61 ± 0.42
MaxD ± SD	16.61 ± 7.55	18.84 ± 9.99	21.82 ± 8.10

**Table 4:** The results of the landmark comparison by describing the mean distances for each landmark between the scanner and the ground truth.

	Polycam (mm)	SP (mm)	EM3D (mm)
g	1.06	1.29	1.59
n	0.82	1.15	1.58
exl	1.45	1.33	1.54
exr	1.6	1.72	1.85
prn	0.92	0.83	1.09
ls	1.13	0.9	1.0
st	1.74	1.65	2.02
li	1.32	1.32	1.13
pog	1.42	1.18	1.23
**Mean**	**1.27**	**1.26**	**1.45**

**Table 5:** The results of the Kruskal–Wallis test between each scanner for every landmark.

	H	*p*
g	4.39	0.111
n	8.72	0.013
exl	2.53	0.282
exr	0.73	0.693
prn	3.57	0.168
ls	2.91	0.233
st	1.09	0.580
li	0.27	0.876
pog	0.53	0.768
**Mean**	**2.75**	**0.414**

## Data Availability

The data that support the findings of this study are available from the corresponding author upon reasonable request.

## Abbreviations

3D	three-dimensional
CBCT	cone beam computed tomography
CT	computed tomography
IQR	interquartile range
MSD	mean surface distance
MaxD	maximal distance
RMS	root mean square
SD	standard deviation
SP	ScandyPro
EM3D	Ethan Makes 3D Scanner
